# Emerging Trends in Atherosclerosis: Time to Address Atherosclerosis From a Younger Age

**DOI:** 10.7759/cureus.56635

**Published:** 2024-03-21

**Authors:** Yazan Almohtasib, Andrew J Fancher, Khalid Sawalha

**Affiliations:** 1 Internal Medicine, University of Missouri Kansas City School of Medicine, Kansas City, USA; 2 Internal Medicine, University of Kansas School of Medicine-Wichita, Wichita, USA; 3 Cardiometabolic Medicine, University of Missouri Kansas City School of Medicine, Kansas City, USA

**Keywords:** cardiovascular disease, younger age, ultra-processed food, cardiometabolic risk factors, atherosclerosis

## Abstract

Over the past two decades, research efforts into cardiovascular disease (CVD) have uncovered findings that fundamentally challenge our understanding of CVD, particularly atherosclerosis. Atherosclerosis was primarily attributed to the well-described abnormal lipid accumulation theory, involving plaque growth with subsequent plaque hemorrhage resulting in acute vessel thrombosis that may or may not rupture. This perspective has now evolved to encompass more complex pathways, wherein the accumulation of abnormal products of oxidation and inflammation is the most likely factor mediating atherosclerotic plaque growth. Furthermore, atherosclerosis was traditionally thought of as a disease in patients aged 40 and older. However, mounting evidence has demonstrated that significant atherosclerosis and CVD events are more prevalent in younger patients than previously realized and accelerating in incidence. With this alarming trend among younger individuals, our review sought to explore why this trend may be happening and what can be done about this developing problem.

## Introduction and background

Atherosclerosis is often assumed to be a disease of older age groups, but this is an incomplete understanding of this disease. Atherosclerosis more frequently begins at young ages and progresses silently as we age [1]. As far back as the First World War, autopsy studies have demonstrated atherosclerotic lesions in the coronary arteries of young men [2,3]. The Bogalusa Heart study was a particularly powerful example of this phenomenon, evaluating 204 individuals aged two to 39 years who had passed away from non-cardiac-related causes. This study found fatty streaks in the aorta of every individual studied and fatty streaks within the coronary arteries of half of the children examined aged two to 15 [4]. Over the last 120 years, it has become increasingly clear to the cardiovascular community that while the most significant consequences of atherosclerosis typically occur later in life, the disease course begins early on.

This is supported by other recent studies that have suggested that even this may be an incomplete understanding of the disease, as atherosclerosis and its consequences are becoming more prevalent in younger age groups. For example, an autopsy study examining 243 cases from 2002 to 2006 found that CAD was the cause of sudden cardiac death (SCD) in 37% of the 21-30-year-old age group and responsible for 80% of deaths in the 31-40-year-old age group [5]. Another investigation into recent global epidemiologic studies from 2012 estimated that between 5% and 10% of adults younger than 40 have experienced myocardial infarction, with approximately 80% attributable to atherosclerosis [6]. A study of 1,635 individuals under 45 years of age without known CAD undergoing coronary computed tomographic angiography (CCTA) demonstrated that one out of five patients had evidence of CAD, with nearly one out of 20 having obstructive CAD [7]. Mounting evidence strongly suggests that atherosclerosis and its consequences are not just confined to patients over the age of 40.

Even more intriguing is that not only do recent studies suggest that atherosclerosis and CAD are pressing issues in younger age groups, but they also indicate that these issues are accelerating in both prevalence and severity. A comparison of two autopsy studies from 1991 to 1992 and 2010 to 2013 demonstrated a significantly higher incidence of atherosclerosis in all age groups, with 68.33% in the older study and 90.83% in the more recent study [8]. The first study found incident rates of CAD at 25% in the 11-20-year-old group, 45.45% in the 21-30-year-old age group, and 75% in the 31-40-year-old age group. The later study showed incidence rates of CAD at 44.44% in the 11-20-year-old age group, 84.61% in the 21-30-year-old age group, and 100% in the 31-40-year-old age group (Table 1) [8]. Another study of individuals presenting with type 1 myocardial infarction found that the proportion of patients with this initial presentation at or below 40 years of age increased throughout 2000-2016, with an average annual increase of 1.7% [9]. While we know that atherosclerosis begins at a young age, these studies have shown that significant atherosclerosis develops earlier in life than previously thought and that the consequences of this disease are manifesting more frequently in younger age groups.

**Table 1:** Comparing the incidence of coronary artery disease between the two studies among different age groups. Author's own work

Study Period	Age Group (years)	Incidence of CAD (%)
1991-1992		
	11-20	25
	21-30	45.45
	31-40	75
2010-2013		
	11-20	44.44
	21-30	84.61
	31-40	100

Pathophysiology of atherosclerosis: key concepts and risk factors

To better understand this issue, it is helpful to review the basic concepts of atherosclerosis. The atherosclerotic disease has well-established cardiometabolic risk factors, considered modifiable through lifestyle changes aimed at preventing further disease burden. A comprehensive grasp of the pathophysiology of these factors in the development of atherosclerotic plaque is crucial for preventing cardiovascular events. The two main processes involved in the pathogenesis of atherosclerosis are cholesterol deposition and chronic inflammation [10]. The genesis of atherosclerosis begins in infants and children as a fatty streak composed of inflammatory cells, including monocyte-derived macrophages and T lymphocytes [11]. Due to the inflammatory process, the deposition of lipids leads to formation of the atherosclerotic plaques and fibrous caps [12]. Several factors play a major role in the pathogenesis of these plaques, including low-density lipoprotein (LDL), obesity, hypertension, and smoking.

LDL has a pivotal role in plaque development, undergoing progressive oxidation and internalization by macrophages, ultimately forming foam cells. During atherosclerotic lesion development, these foam cells experience programmed cell death, or apoptosis, leading to the formation of necrotic foam [12,13]. The necrotic foam serves as a repository for cellular debris and lipids. Later, smooth muscle cell migration and collagen deposition form a fibrous cap [14]. LDL is an essential building block in the pathophysiology of plaque development and thus serum LDL levels are a critical modifiable risk factor.

Obesity is another major risk factor in the United States. Of the population aged 20 and older, 32.8% are overweight, and 39.8% are obese [15]. This risk factor affects numerous metabolic processes by increasing triglyceride (TG) levels, inducing cholesteryl ester-transfer-protein (CETP) to exchange cholesterol esters and TG, causing an increase in LDL levels, and contributing to atherosclerosis [16]. Furthermore, obesity is often characterized as a state of chronic inflammation [15,17]. This state is mediated by a complex interplay between adipose tissue dysfunction, macrophage infiltration, insulin resistance, gut microbiota dysbiosis, oxidative stress, and systemic inflammation [17].

Adipose tissue, or fat cells, plays a crucial role in the body's energy balance. In obesity, adipose tissue becomes dysfunctional, leading to an imbalance in the secretion of adipokines, which are hormones and cytokines produced by fat cells [15,17]. This dysregulation results in increased production of pro-inflammatory cytokines such as interleukin-6 (IL-6) and tumor necrosis factor-alpha (TNF-alpha) [17]. This is further accompanied by an increase in the infiltration of immune cells, particularly macrophages, into adipose tissue. These macrophages contribute to the production of inflammatory mediators, perpetuating a state of chronic low-grade inflammation [15]. To complicate things, the state of hyperglycemia resulting from insulin resistance can further exacerbate inflammation [17,18].

**Figure 1:**
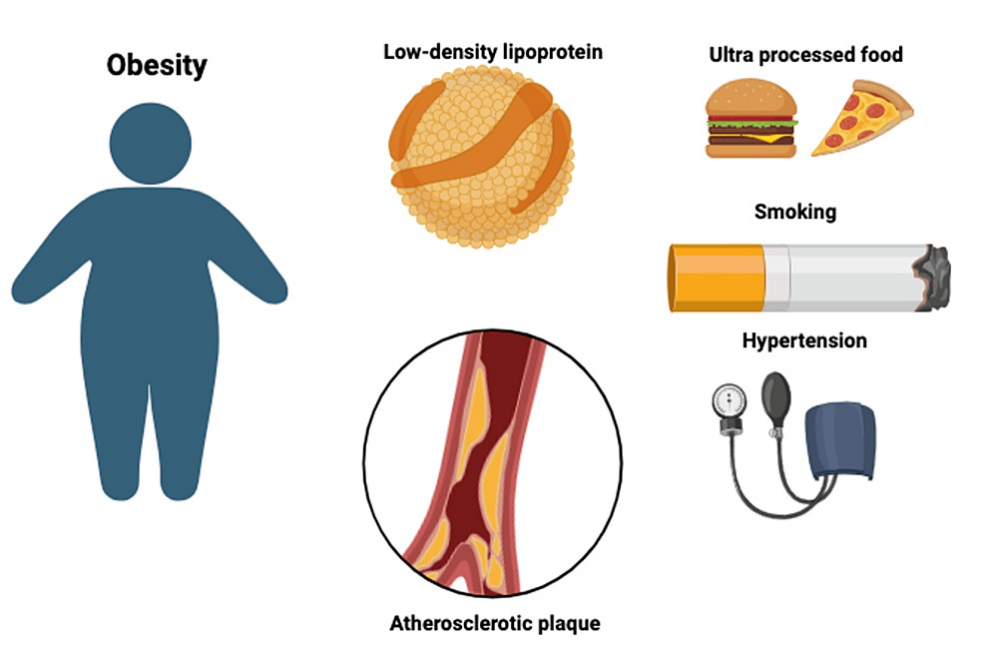
Risk factors associated with developing atherosclerotic plaque in young population. Author's own work

Hypertension is another critical factor in the pathogenesis of atherosclerosis. Hypertension is strongly correlated to the activation of the renin-angiotensin system which can lead to smooth muscle growth, increased inflammation, and the formation of hydrogen peroxide and free radicals such as superoxide anion and hydroxyl radicals in plasma [19]. All of these factors contribute to the development of atherosclerosis through alterations of biochemical processes. Moreover, the physical stress that hypertension causes on the arterial wall itself results in aggravation and acceleration of atherosclerosis (Figure 1) [20].

## Review

Why is this happening in younger age groups?

The natural follow-up question to the findings of increased atherosclerosis and cardiovascular disease (CVD) events in young adults is: why are we seeing significant atherosclerosis and coronary artery disease in younger age groups in recent years? One possible explanation could be the concurrent rising prevalence of common cardiovascular risk factors such as diabetes, obesity, and hypertension in younger age groups. A cross-sectional study of nearly 13,000 adults compared groups from 2009 to 2010 and 2017 to 2020 found an increase in the prevalence of diabetes (3.0% to 4.1%), obesity (32.7% to 40.9%), and hypertension (9.3% to 11.5%) in adults aged 20-44 years [21]. Although a rise in these cardiovascular risk factors helps explain the accelerating prevalence of atherosclerosis and CVD, they still do not identify the root cause.

One possible explanation of that root cause may be changes in our daily diet, particularly an increase in ultra-processed foods (UPFs) consumption. UPFs are industrial formulations that, excluding salt, sugar, oils, and fats, include substances not used in culinary preparations, like additives used to imitate the sensorial qualities of minimally processed foods and their culinary preparations [22]. A cross-sectional study from 2016 found that UPFs comprised 57.9% of Americans' caloric intake and that the content of sugars in these foods was eightfold higher than in processed foods [23]. Ultra-processed foods were first defined by researchers at the Center for Epidemiological Studies in Health and Nutrition at the University of São Paulo, Brazil in the early 2000s, who developed the NOVA classification framework of foods and beverages [24-27]. UPFs have become more and more prevalent in the average American’s diet and may explain some of the trends in atherosclerosis.

A diet high in UPFs is associated with an increased risk of inflammation among other risk factors for CVD. A meta-analysis examining six studies on UPFs, and obesity found a significant association, with a pooled effect size of 1.26, representing 26% increased odds of obesity [28]. An umbrella review of seven systematic reviews examining the connection between UPFs and hypertension performed a meta-analysis demonstrating an odds ratio of 1.23 (95% confidence interval: 1.11 to 1.37, p < 0.001) comparing the incidence of hypertension in high UPFs consumption and low UPFs consumption [29]. A meta-analysis of five studies on connections between type II diabetes mellitus (DM2) and UPFs found that high UPF consumption had a relative risk for DM2 of 1.74 (95% CI: 1.36, 2.22; I2 = 68.9%; p < 0.001) compared to low UPF consumption [30]. A systematic review of UPF consumption and the incidence of dyslipidemia from 2024 found only three studies that met the inclusion criteria. However, their meta-analysis of these three studies showed a relative risk of incidence of dyslipidemia in high UPF consumption of 1.47 (95% CI: 1.12, 1.93), with no significant heterogeneity (I2 = 46%, p = 0.16) [31]. Another study from The Journal of Nutrition followed 13,548 adults aged 45-65 for a median follow-up of 27 years and compared incident rates of CAD between the highest and lowest quartiles of UPF consumption, finding a hazard ratio of 1.19 (95% CI: 1.05, 1.35) [32]. These studies present significant evidence linking the recent increase in UPFs in our diet to the rise in atherosclerosis and CVD events.

High UPF diets may also indirectly contribute to atherosclerosis through alterations in gut microbiota. The gut microbiota, which refers to the diverse community of microorganisms residing in the gastrointestinal tract, plays a crucial role in immune function and inflammation regulation. Obesity and diets high in UPFs are associated with alterations in the composition of the gut microbiota, termed dysbiosis, which can promote inflammation through various mechanisms, including elevated levels of inflammatory markers such as C-reactive protein (CRP) and IL-6 (an indicator of systemic inflammation), and increased oxidative stress that can trigger inflammatory responses and contribute to endothelial dysfunction, immune cell activation, plaque formation, and destabilization [17,18,33,34]. The inflammatory feedback loops in obesity often form self-perpetuating cycles, where inflammation begets more inflammation. This creates a vicious cycle that sustains chronic low-grade inflammation even in the absence of external stimuli promoting atherosclerotic CVD [18,34].

While it is important to analyze the macroscopic effects of UPFs, it is equally important to consider the microscopic impact of UPFs, particularly the epigenetic effects. The field of epigenetics examines how external forces regulate and alter our downstream genetic expression through reversible changes to our genetic DNA sequence [35]. This is a growing area of interest in cardiovascular research, as epigenetic regulations have been demonstrated to play a vital role in coronary atherosclerosis and have been shown to be sensitive to environmental factors, including diet [36].

Ongoing research has identified connections between epigenetic changes and obesity [37-42], atherosclerosis [43-46], and diabetic vascular disease [47,48]. One study by Grimaldi et al. has collected data demonstrating extensive epigenetic changes occurring at many different levels in the pathophysiologic development of atherosclerosis [49]. There is currently limited research on the epigenetic effects of a diet high in UPFs. However, considering the vital role of epigenetics in the development of atherosclerosis, along with evidence strongly suggestive of connections between high UPF diets, CVD, and its risk factors, one must wonder about the epigenetic effects of diets high in UPFs. A possible glimpse into this relationship was found in one study from the British Journal of Nutrition examining the association of UPF intake and urinary levels of 8-hydroxy-2’-deoxyguanosine (8-OHdG), a biomarker of oxidative DNA damage. They found significantly higher levels of this biomarker in the highest of UPF consumers [50]. This is certainly not enough to demonstrate that UPFs create epigenetic changes that manifest as CVD risk factors or CVD, but it does suggest that diets high in UPFs may have more epigenetic effects than we realize.

What can be done?

The second natural follow-up question to the findings of increased atherosclerosis and CVD events in young adults is: what can be done about this? The first place to look for these answers is in our understanding of the prevention and treatment of atherosclerosis in traditionally targeted age groups. As detailed above, many modifiable risk factors in the development of atherosclerosis have been identified and well-researched. Initiatives to improve diet, exercise, and lipid levels have been proven as critical efforts in preventing and treating atherosclerosis.

Recent findings of the DISCO trial (Dietary Intervention to Stop Coronary Atherosclerosis in Computed Tomography) underscore the critical roles of exercise and nutrition in preventing and managing coronary artery disease (CAD). One aspect of the study highlighted the efficacy of High-Intensity Interval Training (HIIT) in inducing CAD regression [51]. These HIIT sessions were structured two times per week, aiming to target 60%-95% of peak heart rate through different intervals with supervision from a physiotherapist with experience in cardiac rehabilitation. The study's endpoints observed the percent atheroma volume (PAV) and total atheroma volume (TAV) through intravascular ultrasound. The results showed a notable 1.4% reduction in plaque volume. While seemingly modest, even slight decreases in plaque can yield substantial benefits, as evidenced by the association between a 1% reduction and a 20% decrease in cardiac events [51].

The effects of diet and lifestyle modification were also observed in the DISCO trial [51]. In this portion of the study, all patients were on optimal medical therapy, and they were randomized into experimental and control groups. The main intervention group followed up with a dietitian to ensure adherence to the dietary approaches to stop hypertension (DASH) model and an increase in physical activity. The atheroma percentage was followed by CT coronary angiography and measured through PAV and TAV. The results showed that the DASH diet did not directly reduce plaque volume. However, it facilitated the transition of non-calcified plaque to calcified forms [52], typically more stable and less prone to rupture, thus lowering the risk of heart attacks [52]. The findings of the DISCO trial strongly outline the synergistic effects of exercise and nutrition in CAD prevention and management, further cementing their role in the prevention and management of atherosclerosis.

Another key strategy is the treatment of atherosclerosis through lipid-lowering agents. Clinical trials such as REVERSAL, ASTEROID, and SATURN have demonstrated the advantages of intense lipid-lowering treatments, showcasing their ability to halt atherosclerotic plaque progression and potentially induce regression [53-55]. These plaques have been observed in a meta-analysis of 31 studies through intravascular ultrasound by measuring TAV and PAV, indicating coronary plaque volume. The study showed TAV was significantly reduced when the LDL levels were less than 80 mg/dL at follow-up, and PAV showed a significant decrease when the LDL levels were less than 90 mg/dL at follow-up [56]. In clinical practice, recent meta-analysis results indicate that in individuals with a five-year risk of major vascular events below 10%, each mmol/L reduction in LDL cholesterol leads to an absolute reduction in major vascular events. Subsequently, growing evidence supports the pursuit of lower LDL targets to further regression of atherosclerotic plaques in primary prevention for CVDs, especially the persistent effect of statin therapy even after being discontinued [57,58].

In 2013, following American College of Cardiology (ACC) guidelines, Dr. Goff introduced the Atherosclerotic Cardiovascular Disease calculator (ASCVD). This tool incorporates factors such as total cholesterol, HDL cholesterol, systolic blood pressure, diabetes, and smoking history. The aim of the calculator is to estimate the risk of cardiovascular events including coronary or stroke death, non-fatal MI, or stroke in the next ten years and to assess the need for statin therapy as primary prevention for atherosclerotic disease [59]. The calculator's development involved pooling data from four cohort studies, including the ARIC (Atherosclerosis Risk in Communities) study, Cardiovascular Health Study, CARDIA (Coronary Artery Risk Development in Young Adults), and Framingham Original and Offspring Study cohorts [60-62]. The individuals included were both white and black aged 18 to 75. However, the calculator was initially validated for ages 40-75 with subsequent validation extended to the Asian population [63]. Current guidelines advocate the initiation of high-intensity statin therapy for primary prevention of atherosclerosis in adults aged 40 to 75 years who have one or more CVD risk factors (i.e., dyslipidemia, diabetes, hypertension, or smoking) and an estimated 10-year CVD risk of 10% or greater when the LDL level is 190 mmol/dL [64,65].

Prevention is better than cure

First attributed to the Dutch philosopher Desiderius Erasmus around 1500, it is now a fundamental principle of our modern healthcare strategies. The need for early intervention cannot be overstated. Initiatives encompassing diet control, regular exercise, smoking cessation, and screening for cardiometabolic diseases are fundamental in mitigating the risk of inflammation-associated conditions. Addressing these factors early in life can pave the way for better long-term health outcomes and lower the burden of chronic cardiometabolic diseases. With the recent evidence demonstrating a rise in significant atherosclerosis and CVD events among young individuals aged 20-40, we believe that addressing these factors even earlier than current guidelines suggest may solve the growing problem of atherosclerosis in young age groups.

An investigative study into the determinants of progression and regression of atherosclerosis by Mendieta et al. demonstrates the importance of earlier intervention in traditional age groups and points us toward a possible solution to the problem of early atherosclerosis. Their study followed the level of atherosclerosis, measured by 3DVUS imaging, in 3,471 patients over six years to analyze what factors contributed to the progression and regression of atherosclerosis. They found that progressors (defined as a 100% increase in plaque burden at a six-year follow-up) had the highest mean LDL-C and SBP values and were more likely to be smokers [66]. They also found that the impact of higher LDL-C and higher SBP on atherosclerosis progression was more marked in younger participants (across strata of increasing age, there was an attenuation in the odds of atherosclerotic progression at six years for every 10-unit increase in LDL-C and SBP, respectively) [66]. These findings not only confirmed the importance of lipid and hypertensive control in the prevention and treatment of atherosclerosis; but also suggested that earlier intervention is more important. This study only looked at adults older than 40. However, with their findings demonstrating that the impact of high LDL and SBP is stronger at younger ages, it is reasonable to extrapolate that the same is true for patients in the 20-40-year-old age range.

Growing evidence is mounting to support earlier intervention efforts. One retrospective review of data from 4,380 patients aged 3-18 followed until ages (20-45) found exposure to risk factors like high total cholesterol, triglycerides, blood pressure, and BMI was predictive of higher atherosclerotic burdens in adulthood [67]. Another investigation using CARDIA (Coronary Artery Risk Development in Young Adults) study data included 4,958 patients (originally enrolled at age 18-30 years) and plotted LDL-C levels vs. age over a median 16-year follow-up period to examine the association between CVD events, the area under LDL-C vs. age curve (cumulative exposure to LDL-C), and slope of the curve (time course of area accumulation). They found that both areas under LDL-C versus age curve and slope (time course of accumulation) were significantly associated with CVD event risk. By analyzing the slope of the curve, they sought to determine if the time course of area accumulation varied by age and found that the same area accumulated at a younger age compared to an older age resulted in a greater CVD event risk increase [68]. One investigation has acted upon these findings and experimented with the initiation of statin therapy in children with familial hypercholesterolemia. This study by Luirink et al. found that initiation of statin therapy in childhood in patients with familial hypercholesterolemia slowed the progression of carotid intima-media thickness and reduced the risk of CVD in adulthood [69].

These studies demonstrate that our current framework for understanding and treating atherosclerosis and CAD, beginning around age 40, may be missing the most critical window for intervention. As highlighted by Mendieta et al., the impact of elevated LDL in the progression of atherosclerosis is more pronounced in younger age groups. This was further supported by Domanski et al.’s study that also showed an association between area under LDL-C vs. age curve and CVD event risk. By waiting to initiate statin therapy until age 40, a patient who develops significantly elevated LDL-C levels in his 20s may be exposed to nearly 20 years of unnecessary, uncontrolled HLD that has a stronger impact on his development of atherosclerosis than those same levels from age 40-60 may have. This hypothetical patient’s physician not only missed 20 years’ time they could’ve been preventing atherosclerosis progression, but evidence from Mendieta et al. and Domanski et al. suggest they may also have missed the most critical window for intervention. More research into the efficacy and safety of statin therapy in adults aged 20-40 is warranted as rates of significant atherosclerosis and CVD events are rising in this age group.

## Conclusions

Significant evidence is mounting demonstrating increased and accelerating prevalence of atherosclerosis and CVD events in younger age groups (20-40 years of age). There has been a concurrent rise in the prevalence of common cardiovascular risk factors such as diabetes, obesity, and hypertension in younger age groups. This may be explained by the increased percentage of the average diet comprised of UPF as diets high in UPFs have been linked to increased inflammation, obesity, hypertension, diabetes, dyslipidemia, gut dysbiosis, dementia, and malignancy. The importance of primary prevention and early intervention for cardiovascular disease in individuals aged 40 and above is firmly established. Initiatives focused on improving diet, lifestyle, exercise, and other cardiovascular risk factors like obesity, diabetes, hypertension, and hyperlipidemia are well-established within the medical community and have been shown to be effective. We believe that current efforts for CVD screening, prevention, and treatment should be extended to patients aged 20 - 40.

The extension of non-pharmaceutical efforts like diet and lifestyle changes could easily be extended to these younger age groups; however, we believe the evidence is building to suggest the need to lower the age cutoff for considering one of our most powerful tools against atherosclerosis: statin therapy. We know that atherosclerosis begins early in life, we know that high LDL-C is a critical factor in the pathophysiologic process, and we know that statin therapy works well to lower LDL-C. By not considering patients younger than 40 for statin therapy, we may be starting to treat atherosclerosis late and missing the most critical window for intervention. Currently, we fight atherosclerosis once it has developed for long enough to manifest macroscopically. By extending initiatives for prevention, screening, and treatment of atherosclerosis to younger age groups, rather than waiting for problems to develop, we could start fighting this disease at its roots.
